# Remarks, Gleanings, and Extracts

**Published:** 1874-04

**Authors:** 


### REMARKS, GLEANINGS AND EXTRACTS

BY LOCAL EDITORS.

*The Therapeutical Value of the Sulphides.*—An excellent article is contributed to the *Lancet*, February 21st, by Prof. Sydney Ringer, on the sulphides of potassium, sodium and calcium. He says:

I wish to call attention to the value of sulphides present in many natural waters, in abscesses, boils and scrofulous sores. The influence of the group on the suppurative process is easily made manifest. Thus when sulphide of potassium or calcium is administered, a thin, watery, unhealthy discharge becomes at first more abundant, afterwards diminishing, and throughout continues thicker and healthier, possessing indeed the characters of "laudable" pus. The condition of the sore improves correspondingly, and its healing is promoted.

Their efficacy may be frequently demonstrated in cases of the following kind. An unhealthy child, from six to twelve months old, suffers from a slight sore throat, perhaps occurring in scarlet fever or measles. The sore throat produces considerable enlargement of the glands behind the angle of the jaw. The swelling, of stony-hardness, may be sufficiently large to interfere with swallowing and to push the head on one side. Suppuration takes place, but is very deep-seated, and for a long time there is neither redness of the skin nor fluctuation, and the pus very slowly makes its way to the surface, so that a fortnight, three weeks, or even a month may elapse before the abscess bursts, or is fit to be opened, when a deep hole is left, with considerable induration around it. The pain and constitutional disturbance are so great that the child sometimes dies; and even if this termination is averted, the deep discharging hole heals very slowly, owing to the indurated and unhealthy state of the adjacent tissues. If a tenth of a grain of sulphide of calcium, mixed with a grain of sugar of milk, is given in such a case every hour or two hours, the results are most striking. The swelling becomes smaller, the pus reaches the surface in four or five days, and when it is evacuated leaves a benign wound which quickly heals. The effects of these remedies are equally conspicuous in mammary abscesses, although in rare instances they appear temporarily to increase the pain, a remark which seems sometimes to hold good with respect to boils. But as a rule the pain is speedily mitigated. Singular to say, I have found these remedies of much less use in forwarding the maturation and expulsion of pus in indolent buboes, but my experience of their use in buboes has been but small.

In boils and carbuncles these remedies yield excellent results. A tenth of a grain of sulphide of calcium, given every two or three hours, generally prevents the formation of fresh boils, while it lessens the inflammation and reduces the area of the existing boils, and quickly liquefies the core, so that its separation is much more speedy, thus considerably curtailing the course of the boil. Where the skin is not yet broken, and the slow-separating core therefore not yet exposed, the sulphides often convert the boil into an abscess, so that on bursting pus is freely discharged, and the wound at once heals. These remedies meanwhile improve the general health, removing that debility and malaise ordinarily so markedly associated with these eruptions. In some cases, however, as in the deep-seated boils and abscesses of diabetes, they are powerless. In carbuncles the sulphides will generally be found equally serviceable, melting, as it were, the core into healthy pus, and so quickly expelling the dead and otherwise slow-separating tissue. In abscesses and carbuncles it is useful to apply belladonna over the inflamed part to reduce inflammation and allay pain. The skin should be thickly smeared with equal parts of belladonna and glycerine, and over this a poultice applied, renewing the belladonna each time the poultice is changed. Poultices, however, being liable to bring out a fresh crop of boils, one of the following plans should be adopted: Smear belladonna ointment some distance round but not over the boil, and then apply a poultice, the greasy application thus protecting the neighboring tissues. Or, still better, apply a belladonna or opium plaster on leather, with a hole the size of the boil around the swelling, and through the opening smear glycerine and belladonna, covering all with a small poultice. The leather plaster efficiently protects the surrounding skin and averts the production of fresh boils.

I have thought it worth while to mention these useful plans of protecting the boil; but it is scarcely necessary to observe that whilst investigating the effects of sulphides, I have employed them alone, or at most sometimes using only a poultice. The good effects of sulphides are conspicuous in certain scrofulous sores not uncommonly seen in children.

The sulphides appear to me to exercise a very beneficial influence in suppurating scrofulous glands in the neck. Here again they hasten the elimination of the pus, and subsequently the cheesy scrofulous matter. After the abscesses have burst, and continued slowly discharging a scanty, unhealthy pus, and when the edges of the sores have become much thickened and indurated, these remedies render the discharge more abundant, thick, creamy and healthy, considerably hasten the evacuation of the scrofulous matter, which prevents the healing of the wound, and at the same time softens the round indurated edges, so that the sore heals much more speedily. If small doses appear to affect these sores but little, larger doses, as half a grain or a grain, should be given several times a day, or even every two hours. I need hardly say that to compass the results described the treatment must be continued several weeks, for it is vain to expect them to occur in a few days, when the sores have been discharging perhaps for months or even years.—*Medical and Surgical Reporter.*

*Spinal Anœmia.*—J., aged 25, had been for three years a sufferer from various nervous and visceral disturbances, and for about two years of that time had been unable to attend to any regular work, owing to the condition of his health. From the patient's account of his symptoms, his disorder had been protean in character, and he had been treated at intervals by different physicians for dyspepsia, heart-disease, cerebral congestion, general debility, etc.

The patient complained of an inability to concentrate his thoughts upon any subject, and of an almost constant feeling of uneasiness in the head, which sometimes seemed constricted, at other times light; but he was very little annoyed with headaches. Disturbance of the stomach was a prominent feature in the case. He was troubled nearly all the time with gastric flatulence and acidity, and a feeling of oppression in the epigastric region, and occasionally with pain in the stomach, and nausea. He was never constipated, but now and then had attacks of diarrhœa, and suffered frequently from abdominal pain and soreness, which he attributed to wind. Palpitations and pain over and about the region of the heart, probably intercostal neuralgia, were among the occasional symptoms. During the greater part of the last two years he had been subject to nocturnal emissions twice and three times weekly. He had difficulty in walking, from a feeling of weakness in the legs and of uncertainty of gait. He always felt better when lying down, and just after arising from the recumbent position. He stated that his mind was always in an irritable condition.

On examination of the spine, tenderness on pressure was found from the third to the tenth dorsal vertebra, most marked over the eight and ninth; and there was tenderness also over the third and fourth lumbar vertebræ. Slight pain in the cord, chiefly in the upper dorsal region, was developed by percussion. The case was diagnosed as one of anæmia of the posterior columns of the spinal cord.

Blisters over the seats of tenderness were ordered to be applied, and repeated as soon as possible. The following prescription was given: ℞. Strychniæ sulphat., gr. j.; ferri pyrophosph., quiniæ sulph., aa. ʒ ss.; acid. phosph. dilut., syrupi zingiberis, aa. f℥ ij.—M.

S.—A teaspoonful to be taken three times daily, immediately after each meal.

The induced current was applied twice weekly to the muscles of the body and extremity. Exercise in the open air, and good nutritious diet, were also enjoined.

At the time of making this report the patient has been three weeks under the treatment just given, the blisters having been twice repeated. The palpitations, neuralgic and other pains, flatulence, acidity, nausea, and nocturnal emissions have ceased to annoy him. He experiences very little uneasiness in the head, and can walk a long distance without difficulty. He expresses himself as feeling better than he has done before for nearly three years. The same treatment will be continued.—*Hospital Report in the Philadelphia Medical Times.*

*Acute Articular Rheumatism.*—Dr. W. H. Farrington, (Clinic Bellevue Hospital, Philadelphia Medical Times) says: The alkaline treatment is the one most generally adopted in the medical wards. A prescription which has found great favor is as follows:

℞. Sodæ bicarb., ℥jss.; potass. acetat., ℥ss.; liq. ammon. acetat., f℥iij.; aquæ, q. s. ad Oj. M. ℞. Acid. citric., ℥ij.; aquæ. Oj. M. ℥ij of each q. 3 vel 4 h. This forms an agreeably effervescing mixture, rendering the urine alkaline very quickly. The frequency with which it is given depends upon the degree of alkalinity obtained as tested morning and evening.

On another division an effervescing draught is made without the addition of the salts of potassa. As a local application, tincture of iodine is sometimes employed. By some the painting is limited to the integument immediately over the joint; by others, the integument corresponding to the blood-supply of the joint, a broad band above and below the joint answering this purpose very well.

Usually, however, cotton dusted with potass. nitrat. and covered by oiled muslin is applied to the joints, affording marked relief. An opiate is given at night: ℞. Sol. morph. sulph. (mag.), ♏lxxx.; tr. belladonnæ, ♏xvj.; aq. fœniculi, ℥ij. M. Sig. ʒj p. r. n. If a friction-murmur develope in the pericardium or pleura, an attempt is made to arrest the inflammatory process by vesication, collodion cum cantharide being used for its purpose. If, however, this fails, hot fomentation are applied to the affected side. Spongio-piline wrung out in hot water and covered with oil silk is generally used. A young girl developed acute pleuritis, left side, and pericarditis, in the course of an attack of rheumatic fever. Spongio-piline applications to the side, changed every two hours, relieved wonderfully the intense lancinating pain she experienced, and appeared to hasten the absorption of the effused fluid. Alkalies were not used in this case. Tr. aconit. rad., ♏j q. 1 h., was given, modifying the amount of febrile disturbance. The patient rapidly convalesced.

In subacute rheumatism, iron, quinine, and cod-liver oil are given internally, with nutritious diet. Occasional revulsives, as tincture of iodine, are applied, as in the acute disease.

In chronic rheumatism, in addition to tonics, a large number of cases have been treated by painting the joint with hydrag. et morph. oleatis, most of them receiving considerable benefit from its use, especially in the alleviation of pain.

*Treatment of Acute Rheumatism by means of Firm Dressing.*—This mode of treatment, recommended by Scutin, and applied in three cases by Gottschalk in 1845, was forgotten, until again brought forward by Concato, and more recently by Heubner. It is the subject of a paper by W. Oehme in the *Archiv der Heilkunde*, whose observations have been made in *Wunderlich's Clinic*. The results of the applications of light bandages are—1. That the pains cease very soon after their application; the application consisting of pasteboard, occasionally, in children and patients in delirium, with a plaster of paris bandage superimposed. As a rule, indeed, the pains cease in from twelve to forty-eight hours, or at latest after seventy-two hours. 2. The febrile symptoms are materially diminished in violence and shortened in duration. In a series of ninety cases, whilst the average duration of the febrile symptoms in the cases treated without the firm dressing, and for the most part with digitalis, was 9.3 days in hospital (plus 4.6 days before admission), those treated with firm dressing had a duration of 6.1 days. A fall of temperature usually occurs after twenty-four or forty-eight hours after the application of the dressing. 3. The combination of the reduction of the pain and of the duration of the fever caused a shortening of the total duration of the disease, and hence acted prophylactically in preventing fixation of the joints. Lastly, the profure sweats characteristic of the disease seem to be diminished.—*Archiv der Heilkunde*, 1873, xiv, p. 385.

*Use of Chlorate of Potash and Glycerine Injections for the Ulcerations in Chronic Dysentery.*—Dr. T. Mead, (*Nashville Medical Journal*) writes: My treatment is, to inject into the bowel half a drachm of chlorate of potash, rubbed up in half an ounce of glycerine, and mixed with three to four ounces of warm water, two or three times a day, the patient to be confined to the bed, with instructions to hold the enema as long as possible. The first injection will not be held over half a minute, and not that length of time if the rectum be much affected. But in a few days the trouble in this respect will be greatly overcome, and, as the ulcerations heal, greater tolerance of the medicine will be evinced. After these injections have been used for from seven to ten days, the whitish membranes and *débris* of the ulcers, provided it be an old case, will be passed with the stools, looking like scraped lint. This occurred in the first case I had, to the no small surprise of my patient. The general health of the patient, of course, needed continued attention, and such medication as the case indicated was prescribed.

*Collodion and Silk Gauze as a Dressing in Mammary Abscess.*— Dr. Douglas Morton, Physician to the Woman's Department of the Louisville City Hospital, has been using for some time back collodion and silk gauze as a compressive dressing in inflammations and abscess of the mammary glands with the most satisfactory results. He writes of it: "Strips of 'Donna Maria' gauze (or a very fine silk barege, such as ladies now use for veils), cut of the requisite length and width and secured by collodion, constitute a dressing in case of inflamed breasts so much superior to that of strapping with the ordinary plaster that I think it merits being more generally known. For some time past I have used no other dressing in breasts requiring support and compression. The manner in which it is applied is sufficiently well known. Among its many advantages may be mentioned that with a little care it can be applied over any well-fitting plaster; and when suitably fenestrated medicines in different forms may be made directly to the parts without affecting the integrity of the dressing."—*American Practitioner.*

*French Experiments with Oatmeal.*—We called attention in a recent number of the JOURNAL to the direct value of oatmeal. We see by an article in *La France Médicale* that M. Dujardin-Beaumitz has been experimenting with it as food for young children. He made use of a jelly prepared by soaking a tablespoonful of the meal in a glass of water for twelve hours, then straining through a sieve, boiling till the whole assumes the consistence of jelly, and adding sugar or salt according to taste. According to analysis, 100 grammes of the meal contain 8.7 grammes of water, 7.5 of fatty matters, 62.5 of starch, 12.2 of nitrogenous matters, 1.5 of mineral substances, and 7.6 of cellulose, dextrine, and loss. Its nutritious value, therefore, as food for children, in regard to nitrogenous or plastic elements, and such as are "respiratory," is analogous to that of human milk, or cow's milk. Besides these, it contains more iron than do most of the ordinary articles of food. Four newly-born infants were fed with the preparation just described, and in every case with satisfactory results. In addition to its qualities as food, it acts efficiently against colic and diarrhœa. It enters into the composition of the *syrup of Luther*, which is said to be much used in Germany. M. Gillette, surgeon of the hospital of Melum, has also given oatmeal "combined with cow's milk," to six children, and finds it to be a valuable food in cases where the natural supply of milk is deficient.

*Uses of Conium.*—Dr. Kennedy, of the College of Physicians, of Dublin, gave the following instances where this drug had been used by him with success: A lady, aged 21, had long suffered from hoarseness and loss of voice. Succus conii was administered, and in a month these symptoms disappeared. Another lady, aged 22, had inflammation of the left ovary, with persistent pain. Extract of hemlock was used, and a rapid recovery followed. A compositor, aged 23, had recurrent attacks of severe abdominal pain. His bowels were regular, and his appetite remained good. There was no trace of lead poisoning. Shooting pains across the abdomen, and into the testes were complained of; and a tumor existed between the liver and the cæcum. Pressure on this tumor caused pain, but its exact nature was doubtful. He was much better after ten days' treatment by hemlock. In general bronchitis in a girl, aged 12, the drug was of the greatest use, rapidly causing certain hectic symptoms to disappear. Dr. Kennedy has used hemlock with success in painful affections, such as rheumatism and neuralgia, in phthisis and hectic, and in some forms of dysuria. Its effects appeared to be tonic and anodyne.

*Inhalations of Muriate of Ammonia in Chronic Affections of the Chest.*—In a recent communication to the *Societe Mecdicale des Hopitaux*, Dr. Libermann described the results which he had obtained through the employment of an inhaling apparatus containing muriate of ammonia, in chronic diseases of the respiratory organs. A description of the apparatus was given, by which it appeared that it was handy, applicable to children, and available for inhalations of a great number of volatile substances. The good results afforded by the drug were attributed to its exciting, irritating action, which produces a revulsive effect. The cases which were especially benefited by muriate of ammonia thus employed included clergymen's sore-throat, in its chronic inflammatory form, and which was rapidly amended, and sometimes speedily healed; chronic bronchitis, which, through its catarrhal element, was invariably amenable to the action of the remedy; and nervous affections of the air-passages, especially convulsive cough and the various forms of idiopathic asthma.—*Lancet.*

*A Simple Plug for Rectal Hemorrhage.*—Two years ago, under somewhat urgent circumstances, Furneaux Jordan, F. R. C. S., devised a plug, which he has found ready, simple, and efficient, in checking hemorrhage from the rectum, whether recurrent or secondary. The tip of the forefinger thrusts the center of a thin white pocket handkerchief well into the rectum; large marbles of compressed cottonwool (or rag) are then gently pushed into the pouch in the rectum, until it is amply distended, and of a balloon shape. Expulsive efforts, or moderate traction on the handkerchief, increase its hæmostatic efficiency. The wool marbles may be moistened with the perchloride of iron, if it be deemed necessary. A plug saves the necessity of pulling down the rectal wall with a vulsellum forceps, and tying the bleeding point—not a pleasant alternative, especially without chloroform. In twenty-four or forty-eight hours the rectum converts the balloon into a cylinder, which is readily withdrawn at pleasure.—*Surgical Inquiries.*

*Albuminuria.*—Nearly thirty yerrs ago Prof. Wm. Darrach used to recommend in albuminuria, and especially in recent cases, small doses (ʒss) of sulphate of magnesia. Dr. Moore, of Rochester, New York (*Phil. Med. and Surg. Report.*), assures us that albuminuria may linger as an organic disease for a considerable length of time and still be amenable to the curative action of sulphate of magnesia. From 20 to 30 grains of the sulphate is given in two ounces of lemonade as often as is necessary to keep the bowels soluble; under this treatment a large percentage of these cases will recover. Another valuable remedy in albuminuria, attended with anæmia and œdema, is to dissolve iron by hydrogen in acetic acid by the aid of gentle heat, and give from 20 to 40 drops in water every 2 to 4 hours. This, in connection with the acetate of potash in 2 to 4 grain doses, and, where the bowels are costive, sulphate of magnesia in 20 to 30 grain doses three times a day, has afforded excellent results.

*Bromide Potassium as a Diuretic.*—Dr. J. B. S. Albyne, (*Medical Archives, St. Louis*) says: So invariable we have found this result, that in very many instances we have attributed a part of the good effects to this very diuretic action. A depletory effect, which, in affections of the head, especially with children, is one of the indications to be met. In certain derangements of the economy, as of simple fever, originating from slight cause, as cold, or digestive disturbance, where almost the sole prominent symptom is an elevated temperature, and the relief to be derived is through the skin or kidneys, following out the above ideas, we have used the bromide as a diuretic simply, and gained very excellent results. Per contra, its use, therefore, would be contraindicated in troubles attended with hypersecretion of urine, or in diabetes. And such seems to be the conclusion which those have reached who have so used it, cases of diabetes being aggravated under its employment.

*Sir James Paget* says the best wash for hardening the skin to prevent bed-sores, is one part of sweet spirits of nitre to three parts of water.

*Acetic Acid in Psoriasis* (New York Med. Jour., Jan., 1874).—Dr. Buck, of Lubeck, says that by ordinary methods of treating psoriasis, the eruption may be temporarily removed without difficulty; but no protection from relapses is afforded. He believes that in nearly all chronic cutaneous eruptions, and especially in psoriasis, an etiological treatment is impossible, and a permanent result is promised only by a consistent external treatment. He has obtained the best results from the use of acetic acid. After the epidermal growths have been softened and loosened by several warm baths and soap, the glistening scales are removed with a soft brush. At first, a few points of the eruption are to be penciled once a day; and this is to be repeated until the skin remains smooth and feels normal to the touch. There are never any scars left. The treatment lasts from four to six or eight weeks, and, properly carried out, is not followed by relapses.—*Phil. Med. Times.*

*How to Check Coughs.*—Dr. Brown-Sequard, in his late Boston lectures, says that there are many facts which show that morbid phenomena of respiration can always be stopped by the influence of arrest. Coughing, for instance, can be stopped by pressing on the nerves of the lip in the neighborhood of the nose. A pressure there may prevent a cough when it is beginning. Sneezing may be stopped by the same mechanism. Pressing in the neighborhood of the ear, right in front of the ear, may stop coughing. It is also preventive of hiccough, but much less so than of sneezing or coughing. Pressing very hard on the top of the mouth inside is also a means of stopping coughing. And, he adds, that the will has immense power there. There was a French nurse who used to say, "The first patient who coughs here will be deprived of his food to-day." It was exceedingly rare that a patient coughed then.—*Medical and Surgical Reporter.*

*Hemorrhoids in Children.*—M. Bouchut writes to the *Gazette des Hôpitaux* that although many children have been brought to him, during the last twenty years, as suffering from hemorrhoids, he has always found that there has been an error of diagnosis, and that they have been the subjects of rectal polypus. Removal of the polypus is easy, and hemorrhage at stool ceases.—*The Obstetrical Journal.*

*Cholera Infantum.*—Dr. Staples (North-Western Med. and Surg. Jour.) thinks highly of ergot in children's diarrhœa. In one case he gave the following: ℞. Squibb's fl. ext. ergot, f ʒiij.; potas. bromidi, ℈ii.; potas. bicarb., gr. x.; aquæ cinnamom; syr. simpl., aa. f ℥iss. M. Signa.—Dose: a teaspoonful once in an hour.

*An Old Cancer Cure Revived.*—Dr. Brewer writes to a recent number of the *Medical Times*, that a patient of his was given by a friend, the following recipe: ℞. Chlor. zinci, gr. viij.; blood root, gr. v.; starch, gr. viij. Make into a paste with honey. "The cancer was at this time nearly as large as a hen's egg. After applying the paste for two weeks, he called to see me. I found it had diminished to half its former size. I advised him by all means to continue it. After a month's use of the remedy the cancer was not larger than a dime. He continued to use it until the disease was cured." This is the "cancer salve" of old Dr. Fell, famous some forty years ago, but long since condemned to neglect.

*Administration of Ether.*—At the Bellevue Hospital, New York, the administration of potass. bromid. gr. xxx, previous, and the same amount immediately following, or as soon as the patient can conveniently swallow, after the administration of sulphuric ether for the purpose of producing anæsthesia, is now regularly resortsd to. The effect is to prevent the vomiting which so commonly follows the use of the anæsthetic.—*Medical Record.*

*Tincture of Iodide of Tannin of Dr. Boinet.*—This preparation is prepared by dissolving 50.0 parts tannin in 500.0 parts distilled water, and adding 25.0 parts tincture iodine. He uses and recommends it as a primary application for all fresh wounds, and states that by this treatment he has never observed diphtheria, and only in isolated cases pyæmia.—*American Practitioner.*

*Remedy for Chronic Hoarseness.*—An eminent physician of Philadelphia contributes the following: In chronic hoarseness arising from thickening of the vocal chords and adjacent membrane, the ammoniated tincture of guaiacum is often a very efficacious remedy. It may be appropriately mixed with equal parts of the syrup of senega, and a teaspoonful given two or three times a day.

*Syrupus Antiasthmaticus.*—Take of syrup of morphia, syrup of chloroform, equal parts. Dose: one tablespoonful when necessary. Note.—Syrup of chloroform is prepared in the following manner: Take of chloroform, 5 drops; alcohol, 30 drops; simple syrup, 1 ounce, and mix. Syrup of morphia contains one grain of sulphate of morphia in 4 ounces simple syrup.

*Churchill's Tincture of Iodine.*—Recrystallized iodine, ℥iiss.; potassium iodide, ℥ss.; alcohol, ℥xij.; water, ℥iv. M. One fluid ounce of this tincture contains seventy-five grains, and is therefore two and one-half times as strong as the tinct. iodinii U. S. P.

TREATMENT OF BILIARY CALCULUS BY CHOLEATE OF SODA.—Schiff admits that these calculi are formed of cholesterin, not because this substance is formed in too great abundance, but because the bile does not contain the principles which maintain it in solution. These are the cholates and choleates of soda and potassa, more than the alkalinity of the bile which dissolves the cholesterin. Schiff therefore advises the administration of eight grains of choleate of soda, to be given twice daily, and increased until "saturation" is indicated by irregularity of the pulse, which becomes slow during repose and accelerated by the least effort. The dose may then be diminished, but not entirely suspended—a considerable time, a week at least, being required for the remedy to produce amelioration of the symptoms.—*Imparziale & Gaz. Heb.*

*Cancrum Oris Treated by Saturated Solution of Iodine.*—Dr. J. G. Miller reports (Kansas City Medical Journal, August, 1873) three cases of cancrum oris successfully treated by tonics and the local application of a saturated tincture of iodine prepared by putting as much iodine into the compound tincture as it would dissolve.—*British Medical Journal.*

*The Witch Hazel as a Hæmostatic.*—Dr. Given (Amer. Pract.) strongly recommends the witch hazel in pulmonary hemorrhage. He gives the fluid extract in teaspoonful doses, every twenty minutes, until the hemorrhage ceases. He gives cases illustrating his views, and they are, so far as they go, satisfactory.

*Nocturnal Muscular Cramps.*—A writer to the *British Medical Journal* says, that if a person subject to this distressing affection will place blocks of wood, six inches high, under each post at the head of his bed, and have his bed made slanting from the head to the foot, he will not suffer from cramps.

*Worm Remedy.*—℞. Podophyllin, gr. j.; santonine, grs. x.; white sugar, ʒj.; to be thoroughly triturated, and divided in twenty powders, of which one may be given morning and night.—*Dr. J. M. Scudder.*

*Aqua Crystallina—Crystal Water.*—Take of bitartrate potassa, 10 parts; sugar, 40 parts; and dissolve in distilled water, 600 parts, filter. This a pleasant beverage in febrile diseases.

*Asthmatic Pastiles*—Take of stramonium-leaves two parts, nitrate of potassa one part. Reduce to a coarse powder, and put up in paper cones weighing half an ounce each.

*Sulphur in Diphtheria.*—In several severe cases of diphtheria sulphur has been employed in this city with gratifying success. A teaspoonful of lac sulphuris is administered thrice a day. The false membrane is swabbed with dilute sulphurous acid, and a general supporting treatment adopted.

*Galega Officinalis as a Lactogenetic.*—Dr. Olfinger recommends the employment of this legumenoid as a stimulant to the secretion of milk in nurses. A great number of facts have convinced him that this agent not only increases the quantity of milk but also improves its quality.—*Clinic.*

*Dr. Tobias' Venetian Horse Liniment.*—According to the analysis of Schaedler, published in the Berlin *Industrie Blätter*, a bottle of this celebrated liniment contains 1 ounce ammonia, half an ounce camphor, 1 ounce tincture of Spanish pepper, 7 ounces alcohol, 2 ounces water.

*Sinapisms.*—In making a mustard-plaster, use no water whatever, but mix the mustard with the white of an egg, and the result will be a plaster which will "draw" perfectly, but will not produce a blister, no matter how long it is allowed to remain upon the part.—*The Medical Brief.*

Phosphorus Mixture.—The *Medical Archieves* gives the following formula: Dissolve a grain phosphorus in six drachms of chloroform, add two ounces of glycerine, and shake well. Dose: a teaspsoonful in water three times a day.

*Cough Mixture in Consumption.*—℞. Mist. glycyrrhizæ comp., 4 ounces; syrup. pruni virg, 2 ounces; quininæ, 32 grains; morphiæ sulphat., 2 grains; elix. taraxaci comp., 2 ounces. M.—Dessert-spoonful every four hours,

